# Telemetric Blood Pressure Assessment in Angiotensin II-Infused ApoE^-/-^ Mice: 28 Day Natural History and Comparison to Tail-Cuff Measurements

**DOI:** 10.1371/journal.pone.0130723

**Published:** 2015-06-18

**Authors:** Christopher M. Haggerty, Andrea C. Mattingly, Ming C. Gong, Wen Su, Alan Daugherty, Brandon K. Fornwalt

**Affiliations:** 1 Saha Cardiovascular Research Center, University of Kentucky, Lexington, Kentucky, United States of America; 2 Department of Physiology, University of Kentucky, Lexington, Kentucky, United States of America; 3 Department of Biomedical Engineering, University of Kentucky, Lexington, Kentucky, United States of America; 4 Department of Pediatrics, University of Kentucky, Lexington, Kentucky, United States of America; University of Buenos Aires, Faculty of Medicine. Cardiovascular Pathophysiology Institute., ARGENTINA

## Abstract

Abdominal aortic aneurysm (AAA) is a disease of the aortic wall, which can progress to catastrophic rupture. Assessment of mechanical characteristics of AAA, such as aortic distensibility, may provide important insights to help identify at-risk patients and understand disease progression. While the majority of studies on this topic have focused on retrospective patient data, recent studies have used mouse models of AAA to prospectively evaluate the evolution of aortic mechanics. Quantification of aortic distensibility requires accurate measurement of arterial blood pressure, particularly pulse pressure, which is challenging to perform accurately in murine models. We hypothesized that volume/pressure tail-cuff measurements of arterial pulse pressure in anesthetized mice would have sufficient accuracy to enable calculations of aortic distensibility with minimal error. Telemetry devices and osmotic mini-pumps filled with saline or angiotensin-II were surgically implanted in male apolipoprotein-E deficient (ApoE^-/-^) mice. Blood pressure in the aortic arch was measured continuously via telemetry. In addition, simultaneous blood pressure measurements with a volume/pressure tail-cuff system were performed under anesthesia at specific intervals to assess agreement between techniques. Compared to controls, mice infused with angiotensin-II had an overall statistically significant increase in systolic pressure, with no overall difference in pulse pressure; however, pulse pressure did increase significantly with time. Systolic measurements agreed well between telemetry and tail-cuff (coefficient of variation = 10%), but agreement of pulse pressure was weak (20%). In fact, group-averaged pulse pressure from telemetry was a better predictor of a subject’s pulse pressure on a given day than a simultaneous tail-cuff measurement. Furthermore, these approximations introduced acceptable errors (15.1 ± 12.8%) into the calculation of aortic distensibility. Contrary to our hypothesis, we conclude that tail-cuff measures of arterial pulse pressure have limited accuracy. Future studies of aneurysm mechanics using the ApoE^-/-^/angiotensin-II model would be better in assuming pulse pressure profiles consistent with our telemetry findings instead of attempting to measure pulse pressure in individual mice.

## Introduction

Abdominal aortic aneurysm (AAA) is a focal and pathologic dilation of the aortic wall, which affects 5–10% of men aged 65–79[1]. Progression to aneurysm rupture is catastrophic with 90% lethality, making AAA the 13^th^ leading cause of death in the United States[2]. Current clinical guidelines recommend surgical intervention for aneurysms above 5.5 cm to prevent rupture, but this metric lacks sensitivity and specificity as smaller aneurysms may rupture and larger aneurysms may remain stable[3]. Rupture is a mechanical failure of the aneurysm in which the internal stress (e.g., from aortic blood pressure) exceeds the tissue strength; therefore, evaluating arterial mechanics in the aneurysm, such as wall stress or distensibility[4], may be a superior rupture assessment. In fact, computer models of aneurysm peak wall stress have shown improved specificity compared to size-based assessments[5,6].

Computational and *ex vivo* modeling studies have provided considerable cross-sectional data on human AAA mechanics[7,8]; however, little is known about the evolution of these properties during early growth and development since most aneurysms are only diagnosed after considerable dilation has already occurred. Hence, several investigators have recently begun to study the mechanics of animal models of AAA[9–11], particularly the angiotensin-II (AngII) infused hypercholesterolemia mouse model[12]. When coupled with high-resolution non-invasive imaging, these models allow for longitudinal analyses of *in vivo* changes in AAA mechanics, such as distensibility, during the early growth and remodeling stages of disease.

One limitation of murine studies is that assessment of arterial blood pressure, which is essential for characterizing the internal loads acting on the aorta, is non-trivial. The two major options for measuring murine blood pressure are implantable telemetry devices or non-invasive volume/pressure tail-cuff systems. Both techniques have considerable trade-offs. Telemetry measurements are highly accurate but are invasive, costly, require considerable skill for precise implantation, and are not compatible with magnetic resonance imaging (MRI). On the other hand, tail-cuff measurements are relatively simple and non-invasive, but are known to have limited accuracy[13–15]. These accuracy concerns are particularly relevant for assessments of arterial pulse pressure, which are needed to quantify distensibility and have never been directly evaluated/validated for the tail-cuff method. We hypothesized that tail-cuff measurements of arterial pulse pressure in anesthetized mice would have sufficient accuracy to enable calculations of aortic distensibility within acceptable error.

To test this hypothesis, the specific objectives of this study were as follows. First, we sought to characterize the natural history and variability of aortic blood pressure in ApoE^-/-^ mice with and without continuous infusion of AngII using implanted telemeters for 28 days. Second, we evaluated the accuracy of aortic blood pressure measurements, particularly pulse pressure, via volume/pressure tail-cuff compared to simultaneous telemetry data. Finally, because mouse imaging requires the use of general anesthesia, we sought to characterize changes in aortic blood pressure, particularly pulse pressure, between conscious and anesthetized states.

## Materials and Methods

Twelve 9-week-old male ApoE^-/-^ mice on a C57Bl/6 background were purchased from Jackson Laboratory (Bar Harbor, ME). They were fed a normal laboratory diet with *ad libitum* access to food and water during 14:10 light:dark environmental cycles. All studies conformed to U.S. Public Health Service policies for the humane care and use of animals. The protocol was approved by the Institutional Animal Care and Use Committee of the University of Kentucky (Protocol number 2013–1108).

### Telemetry Implantation

Telemetry transmitters (Data Sciences International: Model TA11PA-C10; St. Paul, MN) were implanted surgically under isoflurane anesthesia in the carotid artery and advanced to the aortic arch. All efforts were made to minimize suffering. Mice were singly housed in ventilated acrylic cages with nesting material (Nestlets and Enviro-dry) following telemetry implantation. A 10 day recovery period was observed prior to activation of transmitters and the start of data collection. There was one fatality resulting from the surgical implant procedure.

### AngII Infusion

Following 2 days of baseline pressure recording, the 11 remaining mice were randomized to receive either AngII (1,000 ng·kg^-1^·min^-1^; n = 6) or saline (n = 5) via continuous infusion with subcutaneous osmotic mini-pumps (Alzet model: 2004; DURECT Corporation, Cupertino, CA) for 28 days. Pumps were implanted under isoflurane anesthesia, as described previously[12]. All efforts were made to minimize suffering. Telemetry data were collected continuously for the duration of the 28-day infusion.

### Pressure measurements under anesthesia

To quantify changes in pressure between conscious and anesthetized states, anesthesia was induced via inhaled desflurane (6–8% in 1 L/min oxygen). An electric heating blanket was placed on the telemetry receiver platform to maintain body temperature. Respiration was visually monitored. After a 5-minute equilibration period, pressure readings were acquired for 10–13 consecutive minutes. When possible, 20 cycles of volume/pressure tail-cuff data were acquired simultaneously during this interval (Kent Scientific, Torrington, CT). At least 5 passing cycles were required to report a measurement. In this way, measurements under anesthesia were acquired two days prior to pump implantation, and on days 9 (without tail-cuff), 16, and 24 after implantation. The corresponding conscious values were taken as the mean over the rest of the light cycle (excluding the 5.5 hours of the anesthesia experiments) for a given day. Some animals were excluded from the tail-cuff measurements due to development of tail damage such that the cuff could not be placed properly. Furthermore, because of heating issues with experiments on day 9, anesthesia data from this time point were excluded from analysis and two subjects (one from each group) died.

### Distensibility error analysis

Distensibility is the ratio of the percentage area (A) change of the aortic lumen between its maximum and minimum size over the cardiac cycle to the aortic pulse pressure (P). In short:

$$Distensibility\;=\;\frac{\left(A_{max}-A_{min}\right)}{A_{max}∙P_{pulse}}\;=\;\frac{\Delta A}{A∙P}$$

In this study, distensibility was not directly quantified. Instead, the error introduced into its calculation by uncertainty in the approximation of pulse pressure compared to its ‘true’ value for fixed areas was computed:

$$Error\;\left(\%\right)\;=\;\frac{\left|\left(\frac{\Delta A}{{A∙P}_{approximate}}\right)-\;\left(\frac{\Delta A}{{A∙P}_{true}}\right)\right|}{\left(\frac{\Delta A}{{A∙P}_{true}}\right)}∙100$$

With the cancellation of the area terms, this error calculation reduces to:

$$Error\;\left(\%\right)\;=\;\left|{(P}_{true}/P_{approximate})-1\right|∙100$$

### Statistics

Descriptive statistics included mean ± standard deviation, 95% confidence intervals of population means, and ranges of individual subject means, as specified. Statistical comparisons of blood pressure natural history during the experiment (differences between study groups and the interactions of group and time) were made using general linear models (GLM) with repeated measures in SPSS (IBM, Armonk, NY). Additionally, simple comparisons of sample means were performed using either two-sample t-tests or Mann-Whitney tests, as indicated, depending on data normality determined by the Shapiro-Wilk test. For consistency, light cycle telemetry data for the time of day during which the anesthesia experiments were performed (09:00–14:30) were excluded from the analyses. Comparisons of systolic and pulse pressures between conscious and anesthetized states were made using paired t-tests. Comparisons between telemetry and tail-cuff measurements were made using the modified coefficient of variation (CoV) and Bland-Altman limits of agreement[16]. Finally, a stepwise multivariate regression model was constructed in SPSS to determine the independent predictors of a given subject’s pulse pressure as measured via telemetry. The threshold for statistical significance was set at p<0.05 for all evaluations. Bonferroni corrections were made, as appropriate, for multiple comparisons with respect to experimental days.

## Results

### Natural History of Blood Pressure in AngII-Infused ApoE^-/-^ Mice

**Fig 1:**
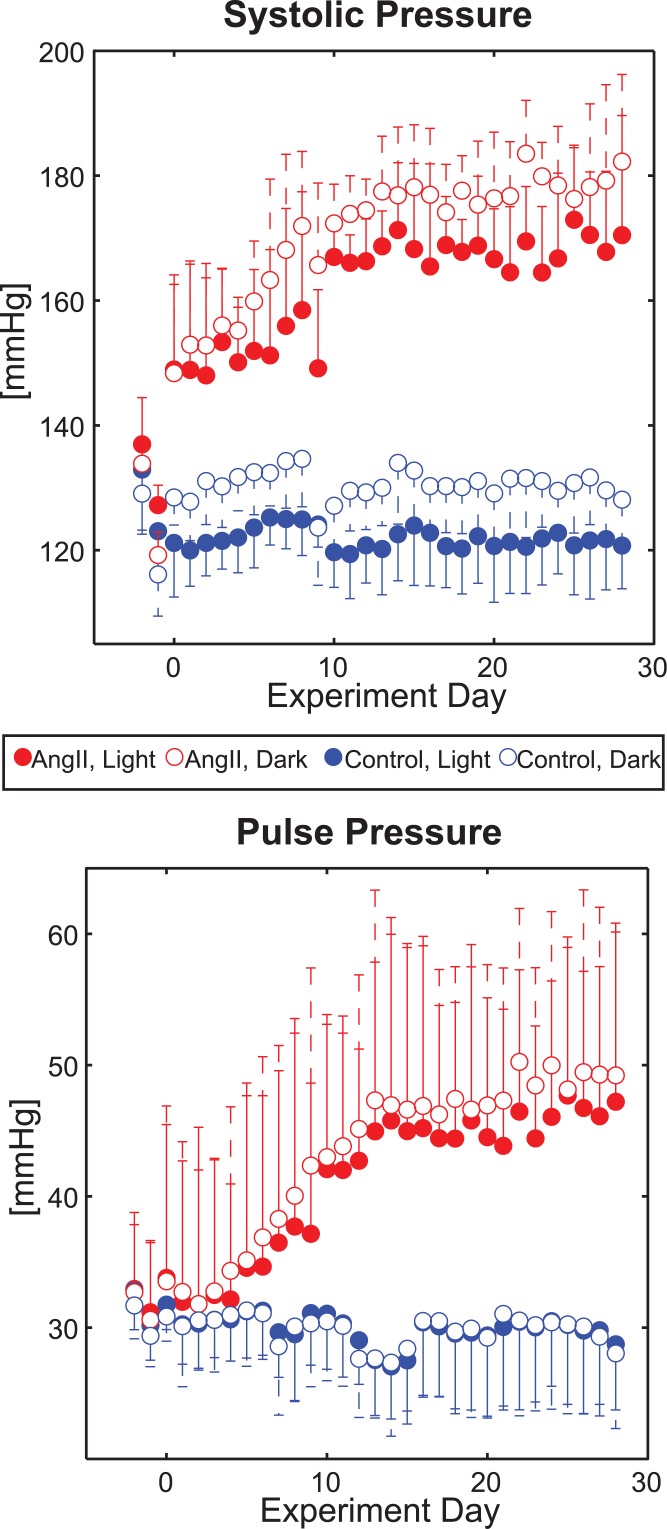
Daily Telemetry Blood Pressure Measurements by Group. Mean and standard deviations for systolic (top) and pulse (bottom) blood pressure via implanted telemetry devices for mice as a function of the number of days they were continuously infused with ‘AngII’ (red) or saline (‘Control’; blue). The filled circles represent data recorded during the light cycle; empty circles report data from the dark cycle.

Mean systolic and pulse pressure telemetry data for each day are shown in Fig 1 for both the light cycle and dark cycle. The raw data for these calculations are stored in Matlab (.mat) files in S1–S12 Files.

Systolic pressure in the AngII group increased by 23% within hours of pump implantation, with Day 0 light cycle means of 149 ± 14 vs. 121 ± 9 mmHg for AngII vs. saline (control) groups, respectively (p = 0.003 by two-sample t-test). From there, mean AngII systolic pressure generally continued to increase daily until it reached a plateau around day 10 (light cycle mean for days 10–28: 168 mmHg, see Table 1). Conversely, daily mean systolic pressure for the controls during the light cycle ranged between 120–125 mmHg for the duration of the experiment. As a result, there was an overall statistically significant group-wise difference in light cycle systolic pressure and a statistically significant interaction of time*group (both p < 0.001 by GLM).

**Table 1:** Descriptive Statistics for Telemetry Blood Pressure Measurements over Time.

		Mean	95% Confidence Interval of group mean	Range of subject means
Systolic Pressure [mmHg]	AngII, Light (days 10–28)	168	158–178	146–193
	AngII, Dark (days 10–28)	177	168–186	159–200
	Control, Light (all)	122	115–130	108–146
	Control, Dark (all)	130	123–138	107–146
Pulse Pressure [mmHg]	AngII, Light (all)	39	27–51	23–71
	AngII, Dark (all)	41	28–53	24–75
	Control, Light (all)	30	26–35	21–38
	Control, Dark (all)	30	25–35	20–38

**Fig 2:**
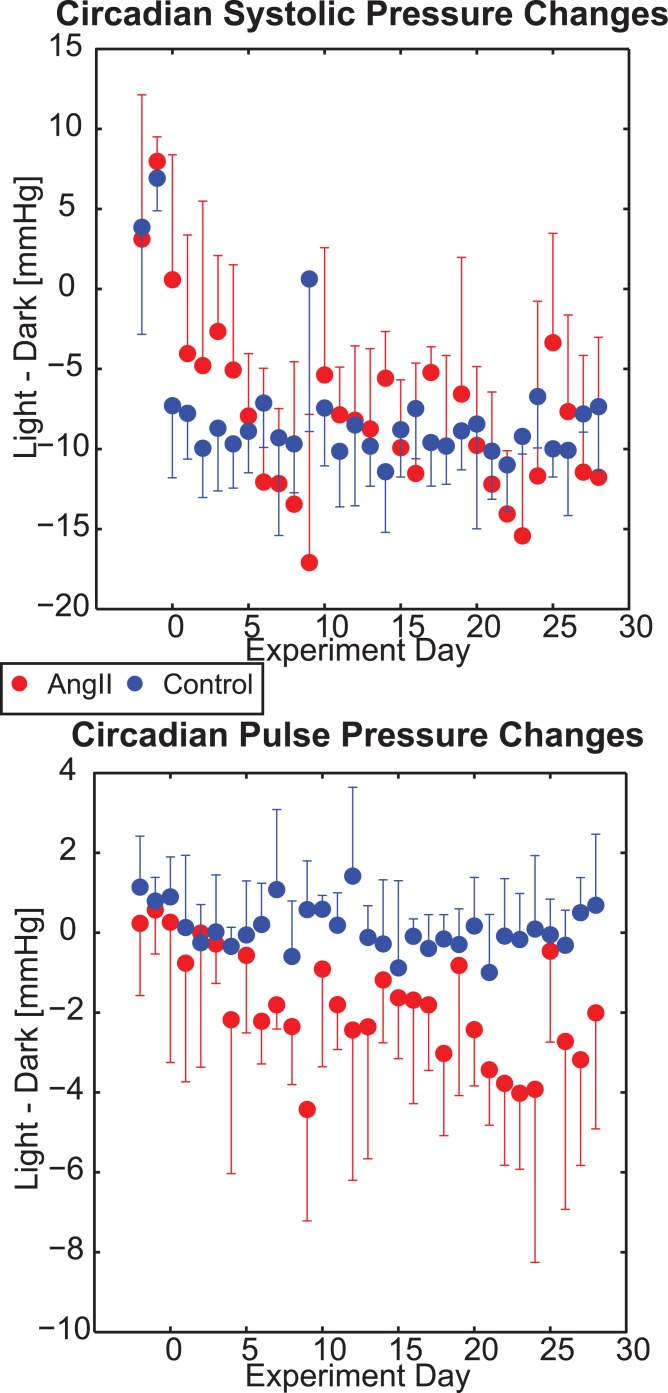
Circadian Changes in Blood Pressure Measurements by Group. Mean and standard deviations of individual subject differences in systolic (top) and pulse (bottom) blood pressure between the dark and light circadian cycles via implanted telemetry devices for mice as a function of the number of days they were continuously infused with ‘AngII’ (red) or saline (‘Control’; blue).

Systolic pressures during the dark (active) cycle were generally higher than the light cycle (Fig 1, Table 1); however, they qualitatively followed similar temporal trends (with respect to the experimental course) as the light cycle for both groups. There was no significant difference in the mean change of systolic pressure from dark cycle to light cycle between groups (-8 ± 3 vs. -8 ± 2 mmHg for AngII vs. controls, respectively, p = 0.85 by t-test; Fig 2).

Mean pulse pressure was qualitatively no different between AngII mice and controls through the early course of infusion, indicating that early changes in diastolic pressure for the AngII mice were consistent with those of systolic pressure. In fact, no overall statistically significant group-wise difference in pulse pressure during the light cycle was observed (p = 0.101 by GLM, Table 1). However, later increases in systolic pressure for the AngII group increased the mean pulse pressure, which resulted in a statistically significant interaction of time*group (p < 0.001 by GLM). A statistically significant difference was first observed between groups on day 11 (using Mann-Whitney tests at the p < 0.05 level) and remained most days thereafter, although a Bonferroni adjustment for multiple comparisons removed these differences.

Additionally, while the qualitative trends in pulse pressure within groups were the same during the dark cycle as the light cycle (Fig 1), a statistically significant circadian difference in pulse pressure magnitudes was seen across groups. Specifically, the AngII group had a larger change in mean pulse pressure from the dark cycle to the light cycle compared to the controls (-2 ± 1 vs. 0 ± 1 mmHg, p = 0.002 by t-test; Fig 2).

### Blood Pressure via Telemetry in Conscious vs. Anesthetized States

**Fig 3:**
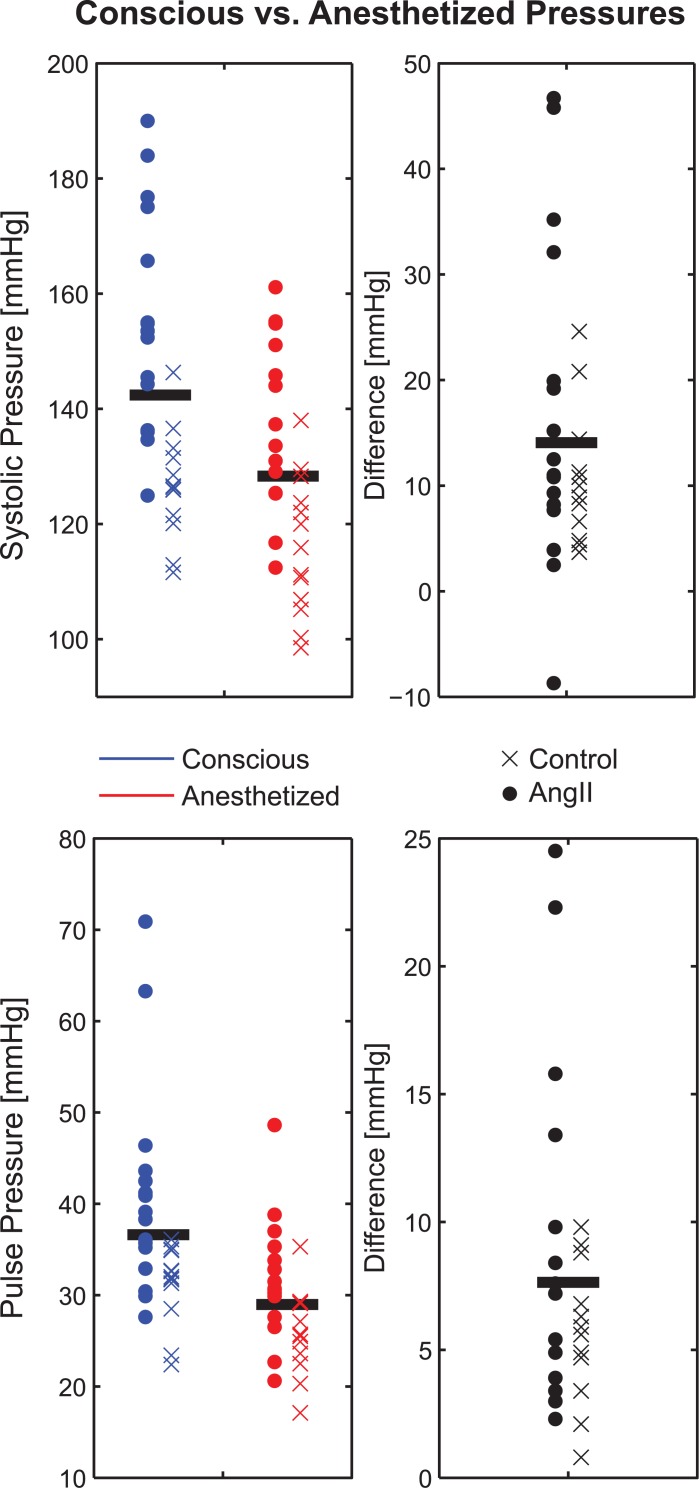
Comparison of Conscious and Anesthetized Blood Pressures. *Left Panels-* Scatterplot comparison of systolic (top) and pulse (bottom) arterial pressure measurements via implanted telemetry under conscious (blue) and anesthetized (red) conditions. Horizontal bars represent the measurement mean. *Right panels-* Scatterplot of the differences between conscious and anesthetized measurements on a subject-by-subject basis. Circles denote subjects infused with AngII while ‘x’s denote controls. Horizontal bars represent the measurement mean.

A comparison of systolic and pulse pressures between conscious and anesthetized states (n = 29 total observations), including paired differences, is shown in Fig 3. Arterial pressure for all subjects decreased under anesthesia (p < 0.001 for both systolic and pulse) compared to conscious values. The mean difference in systolic pressure was 14 mmHg, while the mean difference in pulse pressure was 8 mmHg. These responses were not dependent on group as t-tests of difference vs. group were not statistically significant (p = 0.57 for systolic, p = 0.26 for pulse pressure). See S14 File for original data.

### Comparison of Telemetry and Tail-cuff Results

**Fig 4:**
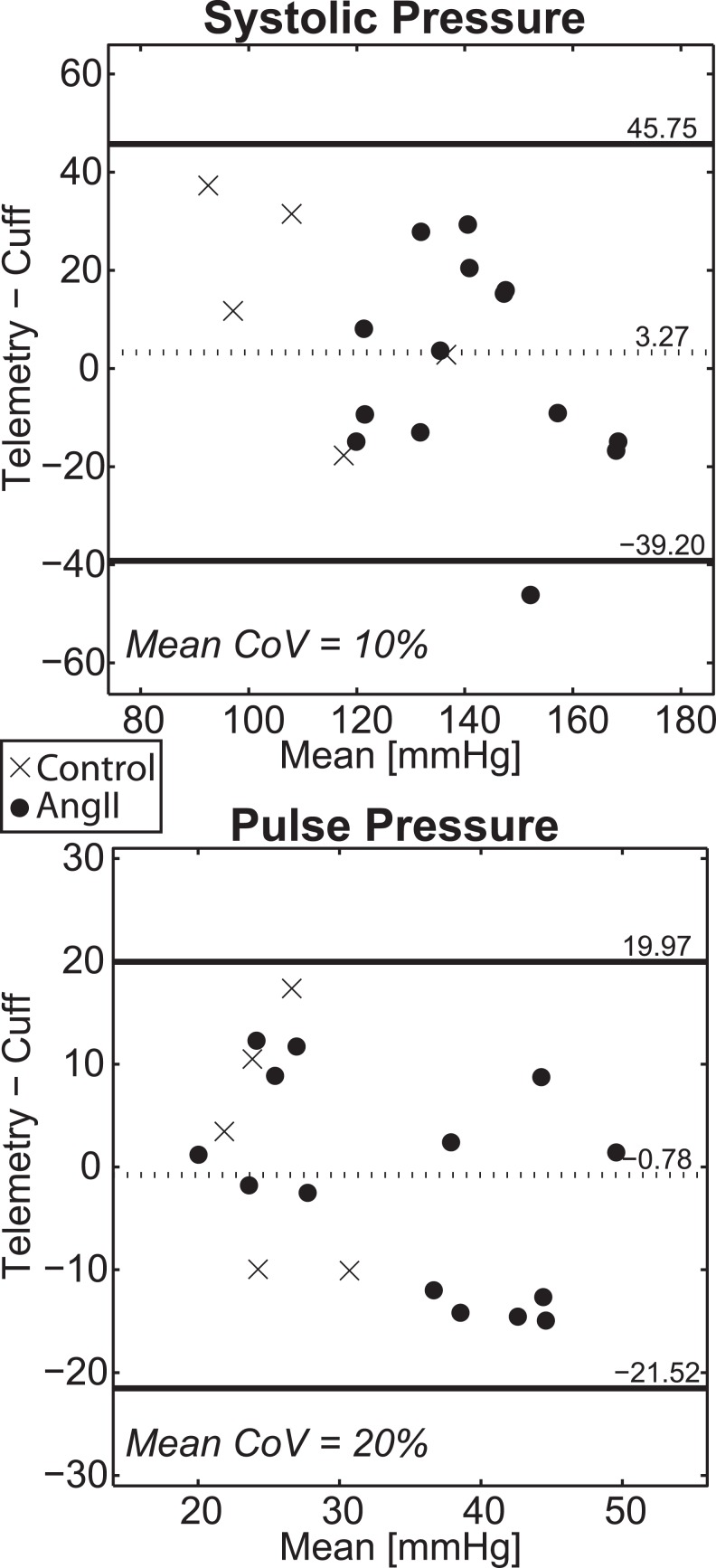
Comparison of Simultaneous Measurements of Implanted Telemetry and Non-Invasive Tail-cuff. Bland-Altman plot comparing telemetry and tail-cuff measurements of systolic (top) and pulse (bottom) blood pressures. Circles denote subjects infused with AngII while ‘x’s denote controls. Overlaid numbers represent the measurement bias (adjacent to the dotted lines) and 95% limits of agreement (adjacent to solid lines). The mean coefficient of variation (CoV) is also reported.

Bland-Altman plots comparing systolic and pulse pressure measurements between telemetry and tail-cuff (n = 19 total observations) are shown in Fig 4. For systolic pressure, there was very good agreement between techniques with mean CoV = 10%. Furthermore, there was a negligible bias (3 mmHg) and the 95% limits of agreement ([–39, 46] mmHg) were small compared to the mean pressure. For pulse pressure, there was a weaker agreement between measurements, with mean CoV = 20%. There was a negligible bias (-1 mmHg), although the 95% limits of agreement ([–22, 20] mmHg) were large compared to measurement means. See S13 File for original data.

**Fig 5:**
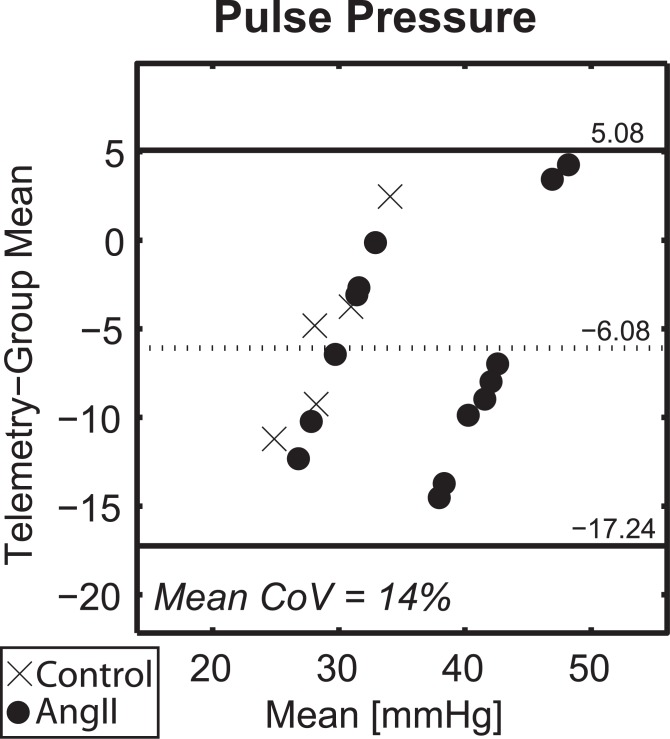
Comparison of Individual Pulse Pressure Measurements under Anesthesia to Conscious Group Average. Bland-Altman plot comparing individual telemetry assessments of pulse pressure under anesthesia and the corresponding group averaged conscious pulse pressure for the same day. Circles denote subjects infused with AngII while ‘x’s denote controls. Overlaid numbers represent the measurement bias (adjacent to the dotted lines) and 95% limits of agreement (adjacent to solid lines). The mean coefficient of variation (CoV) is also reported.

Based on these accuracy findings, we needed to assess the usefulness of tail-cuff arterial pulse pressure measurements for future studies of aortic distensibility. A stepwise multivariate regression model was evaluated in which the individual subject’s telemetry-based pulse pressure under anesthesia was treated as the dependent variable and the independent variables were the corresponding simultaneous tail-cuff measurement for each subject and the ensemble average conscious pulse pressure for the subject’s group for that given day. Based on this model, only the group mean pulse pressure was a significant predictor of an individual subject’s anesthetized pulse pressure value (R^2^ = 0.55, β = 0.74, p < 0.001). In fact, Fig 5 demonstrates that the CoV (14%) and Bland-Altman limits of agreement ([-17, 5]) associated with comparing group-averaged conscious pulse pressure with an individual subject’s telemetry average under anesthesia were considerably smaller than the corresponding comparison of telemetry and tail-cuff measurements (Fig 4). Furthermore, if the bias associated with conscious vs. anesthetized pulse pressure values (7.6 mmHg) is subtracted from the group mean measurements, the CoV is further reduced to 11%. These data led us to reject the stated study hypothesis by demonstrating that tail-cuff pulse pressure measurements are less accurate than estimates from averaged telemetry data.

### Error analysis for distensibility calculation

**Fig 6:**
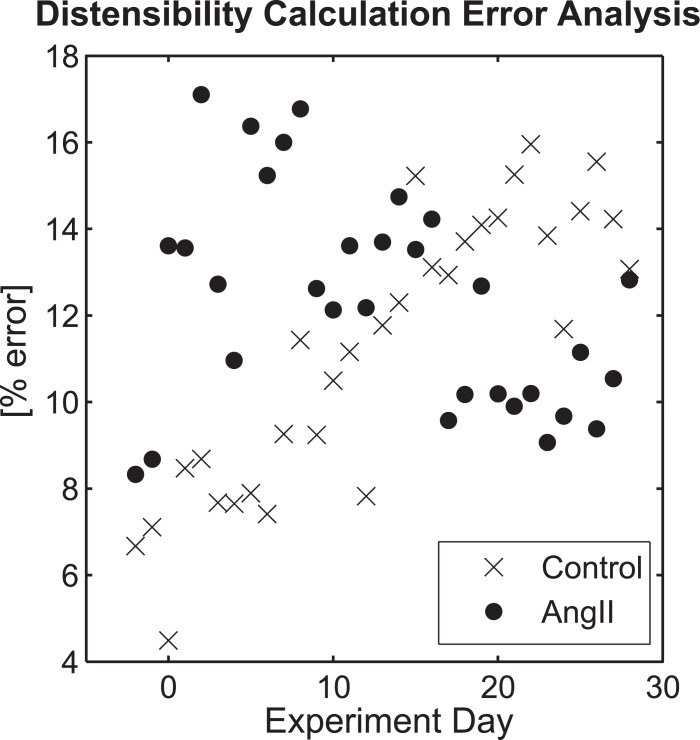
Error Analysis of Pulse Pressure Estimation for Distensibility Calculations by Group. Mean group errors for the calculation of aortic distensibility introduced by the group-averaged estimation of arterial pulse pressure as a function of the time (days) with continuous infusion of AngII (after removal of statistical outlier) or saline (‘Control’).

Group-mean pulse pressure was found to be a better approximation of telemetric pulse pressure data than individual tail-cuff measurements; however, the uncertainty of aortic distensibility calculations based on this mean approximation is still unknown. To address this gap, we conducted an error analysis to determine how the variability in pulse pressure around the group mean would impact calculations of distensibility for a given change in area. The actual calculations of distensibility were not made since imaging studies were not performed. The AngII group had errors of 20–30% for much of the study, particularly during the early course of infusion (overall mean = 19.2%). However, one subject, whose pulse pressure was routinely more than two standard deviations above the group mean (mean pulse pressure for that subject = 61 mmHg), skewed these values higher. With this outlier removed (Fig 6), the mean group error was reduced to 12.3%, which is comparable to the mean error for the control group (11.2%).

## Discussion

Murine models of AAA have the potential to provide novel insights into the evolution of aortic mechanics during the early growth and progression of aneurysms. Such knowledge is lacking from human disease because most human aneurysms have already progressed to a late stage at diagnosis. A primary challenge in leveraging mouse models in this regard is that the accurate quantification of arterial blood pressure in mice, which is a critical element in the assessment of aortic mechanical characteristics, is non-trivial. Hence, the time course and ability to accurately measure arterial pulse pressure, to our knowledge, have never been evaluated in the AngII-infused ApoE^-/-^ model.

The results of this study provide several important insights to address these knowledge gaps. We conducted a direct comparison of telemetry blood pressure measurements to simultaneous tail-cuff data to demonstrate that systolic blood pressure tail-cuff measurements agree well with telemetry. The limits of agreement from these data ([-39.20 45.75] mmHg) agree very well those reported by Feng *et al*. ([-44.25 44.75] mmHg)[14]. However, tail-cuff pulse pressure measurements in anesthetized animals appear to be less accurate with respect to telemetry.

We reported systolic and pulse blood pressure in ApoE^-/-^ mice with and without continuously infused AngII to define normative values during a standard 28-day experimental time course. The potential value of these data, particularly pulse pressure, was demonstrated by showing that the group averaged values provided a better approximate of a given subject’s pulse pressure compared to a tail-cuff measurement. Furthermore, the errors introduced into distensibility calculations by this approximation were acceptably low compared, for example, to the changes and variability in aortic peak cyclic strain, which is related to percentage area change, in response to AngII infusion as reported by Goergen *et al*.[11]. These results suggest that it is better to assume a value for arterial pulse pressure from high fidelity, group averaged data, such as what we report, than to attempt to measure it with a tail-cuff for a given subject. This finding has meaningful ramifications for future murine studies evaluating aortic mechanics of aneurysms.

Finally, we quantified the change (i.e., decrease) in systolic and pulse pressures resulting from inhaled desflurane compared to the conscious state. Understanding these changes has practical significance for any pressure/load sensitive measurements taken from these animals under desflurane anesthesia, such as for non-invasive imaging[10,17].

This study was limited by the use of only a few animal subjects; however, these group sizes are consistent with previous studies reporting blood pressure using radiotelemetry[18,19]. The inclusion of more mice may have allowed for the evaluation of additional variables of interest, such as the effect of high-fat diet. However, the lack of these additional groups and variables did not preclude the evaluation of the primary study hypothesis that tail-cuff measurements of arterial pulse pressure in anesthetized mice would agree with simultaneous telemetry data. Body temperature was not routinely monitored during measurements under anesthesia, which could have additional effects on systemic hemodynamics. Instead, subjects were kept on an electric heating blanket, which was assumed to maintain a physiologic core temperature. Finally, the use of desflurane instead of isoflurane to induce anesthesia for the tail-cuff measurements may result in small physiologic differences compared to procedures performed using isoflurane. Desflurane was chosen to maintain consistency with our current protocol for MRI in mice[20].

In conclusion, this study has defined normative values for arterial blood pressure (both systolic and pulse) in ApoE^-/-^ mice using telemetry with and without continuous infusion of angiotensin II. These data may be of value to future studies seeking to use this model to study aortic mechanics in the setting of abdominal aortic aneurysms. Specifically, given the weak agreement of volume/pressure tail-cuff measurements of arterial pulse pressure compared to telemetry, we propose that future studies should use these group-averaged pulse pressure data in determining the physiologic loads imposed on the murine aortas.

## Supporting Information

S1 File Mean telemetry data for all subjects. (ZIP)

S2 File All telemetry data, AngII subject 1. (ZIP)

S3 File All telemetry data, AngII subject 2. (ZIP)

S4 File All telemetry data, AngII subject 3. (ZIP)

S5 File All telemetry data, AngII subject 4. (ZIP)

S6 File All telemetry data, AngII subject 5. (ZIP)

S7 File All telemetry data, AngII subject 6. (ZIP)

S8 File All telemetry data, Control subject 1. (ZIP)

S9 File All telemetry data, Control subject 2. (ZIP)

S10 File All telemetry data, Control subject 3. (ZIP)

S11 File All telemetry data, Control subject 4. (ZIP)

S12 File All telemetry data, Control subject 5. (ZIP)

S13 File Telemetry and tail-cuff comparison data. (ZIP)

S14 File Conscious vs. Anesthetized telemetry comparison data. (ZIP)
